# Effect of incremental proportions of *Desmanthus* spp. in isonitrogenous forage diets on growth performance, rumen fermentation and plasma metabolites of pen-fed growing Brahman, Charbray and Droughtmaster crossbred beef steers

**DOI:** 10.1371/journal.pone.0260918

**Published:** 2022-01-04

**Authors:** Felista W. Mwangi, Benedicte Suybeng, Christopher P. Gardiner, Robert T. Kinobe, Edward Charmley, Bunmi S. Malau-Aduli, Aduli E. O. Malau-Aduli

**Affiliations:** 1 Animal Genetics and Nutrition, Veterinary Sciences Discipline, College of Public Health, Medical and Veterinary Sciences, Division of Tropical Health and Medicine, James Cook University, Townsville, Queensland, Australia; 2 CSIRO Agriculture and Food, Private Mail Bag Aitkenvale, Australian Tropical Sciences and Innovation Precinct, James Cook University, Townsville, Queensland, Australia; 3 College of Medicine and Dentistry, Division of Tropical Health and Medicine, James Cook University, Townsville, Queensland, Australia; Tokat Gaziosmanpasa Universitesi, TURKEY

## Abstract

Desmanthus (*Desmanthus* spp.), a tropically adapted pasture legume, is highly productive and has the potential to reduce methane emissions in beef cattle. However, liveweight gain response to desmanthus supplementation has been inconclusive in ruminants. This study aimed to evaluate weight gain, rumen fermentation and plasma metabolites of Australian tropical beef cattle in response to supplementation with incremental levels of desmanthus forage legume in isonitrogenous diets. Forty-eight Brahman, Charbray and Droughtmaster crossbred beef steers were pen-housed and fed a basal diet of Rhodes grass (*Chloris gayana*) hay supplemented with 0, 15, 30 or 45% freshly chopped desmanthus forage on dry matter basis, for 140 days. Varying levels of lucerne (*Medicago sativa*) hay were added in the 0, 15 and 30% diets to ensure that all diets were isonitrogenous with the 45% desmanthus diet. Data were analyzed using the Mixed Model procedures of SAS software. Results showed that the proportion of desmanthus in the diet had no significant effect on steer liveweight, rumen volatile fatty acids molar proportions and plasma metabolites (*P* ≥ 0.067). Total bilirubin ranged between 3.0 and 3.6 μmol/L for all the diet treatments (*P* = 0.67). All plasma metabolites measured were within the expected normal range reported for beef cattle. Rumen ammonia nitrogen content was above the 10 mg/dl threshold required to maintain effective rumen microbial activity and maximize voluntary feed intake in cattle fed low-quality tropical forages. The average daily weight gains averaged 0.5 to 0.6 kg/day (*P* = 0.13) and were within the range required to meet the target slaughter weight for prime beef markets within 2.5 years of age. These results indicate that desmanthus alone or mixed with other high-quality legume forages can be used to supplement grass-based diets to improve tropical beef cattle production in northern Australia with no adverse effect on cattle health.

## Introduction

Australia is a major global beef producer. In 2019, Australia was the second-largest beef and veal exporter after Brazil and accounted for 14% of total global beef export. The northern region of Australia encompassing parts of three states including Queensland, Northern Territory and Western Australia accounted for 45, 9 and 8% of the Australian national cattle herd, respectively [1, 2]. Beef cattle are routinely backgrounded on extensive grazing systems and finished on pasture for the lean beef market, or energy-dense grain-fed prime beef markets [1, 3]. Northern Australian tropical beef cattle rely mainly on native grass with few sown grass and legume pastures. In these summer rainfall-dominant dry tropics and sub-tropics, cattle are able to selectively graze in the early wet season, but often lose body condition, experience slow growth and struggle to attain maintenance weight in the other seasons due to low diet crude protein (CP) and digestible energy, pasture senescence, frost and overall poor pasture quality [4–7].

Augmenting grass pastures with legumes has been reported to improve diet CP and energy digestibility [8]. Besides, legumes improve the yield and nutritive value of grass-based pastures since the nutritive value of the resultant diet is higher compared to grass-only diet [9, 10], with specifically profound effects in winter and spring [11, 12]. In northern Australian light-textured soils, tropical pasture legumes came into general use after 1960 [8, 12, 13]. Benefits on the heavier textured soils (the Vertosols) are being evaluated after the development of suitable legumes [14]. Among the new legume pastures developed is desmanthus, a legume native to the Americas. Desmanthus is reported to have the potential for use as a forage legume in extensive grazing systems and crop rotations [8]. Two studies examining growth performance of livestock fed desmanthus-grass diet reported higher liveweight gain in cattle [15] and goats [16] compared to *Cenchrus ciliaris* (buffel) and *Brachiaria mulato* (mulato) grass only diets. In contrast, growing goats fed *Sorghum bicolor* (sudan grass) and supplemented with *D*. *bicornutus* leaves gained less weight than those supplemented with *Leucaena leucocephala* (leucaena), *Medicago sativa* (lucerne) or *Lablab purpureus* (lablab) [7]. Beef cattle supplemented with incremental *D*. *leptophyllus* or *D*. *bicornutus* levels up to 31% dry matter (DM) had similar weight gain with their counterparts fed Rhodes grass (*Chloris gayana*) only diet, although desmanthus supplementation improved rumen fermentation [17]. These studies indicate that there are discrepancies and inconsistencies in animal growth in response to supplementation with desmanthus. Therefore, more studies are required to determine desmanthus’ effect on beef cattle growth performance and change in rumen and plasma metabolites. Our previous study [18], demonstrated that in an extensive grazing system typical of Central Queensland, Brigalow region, backgrounding 400 beef cattle steers during the dry season for 147 days on buffel grass alone or buffel-desmanthus mixed pastures with desmanthus accounting for 11.5% pasture biomass, did not produce any significant differences in liveweight, daily weight gain and plasma metabolites. The lack of difference was thought to be due to similar dietary crude protein levels for steers on both paddocks accessed through browsing on shrubs and forbs, necessitating the need for feeding steers isonitrogenous diets with varying levels of desmanthus inclusion in a controlled pen trial. Therefore, the aim of the current study was to evaluate the effect of increasing levels of desmanthus in isonitrogenous diets on beef cattle growth rate, rumen fermentation and plasma metabolites of tropical crossbred beef cattle. We hypothesized that cattle fed isonitrogenous diets supplemented with incremental levels of desmanthus would have similar growth rates, rumen fermentation and plasma metabolites concentration.

## Materials and methods

This study was carried out at the CSIRO Lansdown Research Station, Queensland, Australia (19.59° S, 146.84° E) between March and July 2020. The station receives 861 mm mean total annual rainfall with mean annual min and max temperatures of 16.8 and 26.1°C, respectively [19, 20]. All procedures in this study followed the CSIRO Animal Ethics Committee approved guidelines (approval number 2019–38) and the Australian code of practice for the care and use of animals for scientific purposes [21].

### Forage establishment and management

Soil analysis was carried out in a 12 ha plot of land before forage establishment to examine suitability of the plot for desmanthus production. Pure stands of three desmanthus cultivars, namely *D*. *virgatus* cv. JCU2, *D*. *bicornutus* cv. JCU4 and *D*. *leptophyllus* cv. JCU7 (Agrimix Pastures Pty Ltd, Ferny Hills DC, QLD, Australia), were planted on 29 November 2019 in 4 ha plot per cultivar at a sowing rate of 2 kg/ha. The plots were irrigated as follows; 15–22 mm/m^2^ every two days for the first 10 days, every three to four days from day 11 to 30 and once a week onwards. Each cultivar plot was divided into three and harvesting was staggered between them to ensure similar maturity stage of the forage at harvest. Overgrown forage was slashed and used as mulch to encourage new regrowth. Plots were not fertilized during desmanthus establishment but were top dressed with Natramin S (4.7% Ca, 0.07 P, 6.3S, 2.8 K, 2.3 Mg, 23.3 Si, 5.1 Fe, 3255 ppm C, 930 ppm Mn, 140 ppm Zn, 56 ppm Cu, 23 ppm Co, 18 ppm B and 6 ppm Mo; Ag Solutions, Gympie, QLD, Australia) at 400 kg/ha in February and urea at 100 kg/ha in March 2020. Weeds were controlled by spraying the plots with 3 L/ha glyphosate-based herbicide (Roundup; Monsanto, Kilda Road, Melbourne, Australia) mixed with 0.7 L/ha 2,4-dichlorophenoxyacetic acid (Titan amine; Titan Ag Pty Ltd, Princes Street NSW, Australia) four weeks pre-planting. Fluazifop-P (Fusilade Forte; Sygenta Australia Pty Ltd, Lyonpark Road, NSW, Australia) and clethodim (Select; Arysta Life Science Australia Pty Ltd, Hindmarsh Square, Adelaide, South Australia, Australia) were sprayed at 0.8 and 0.4 L/ha, respectively, three weeks after planting and twice more during the trial when forage was slashed back to prevent competition from grasses. All herbicides were mixed in 150 L/ha of water.

### Animal management and diets

**Fig 1:**
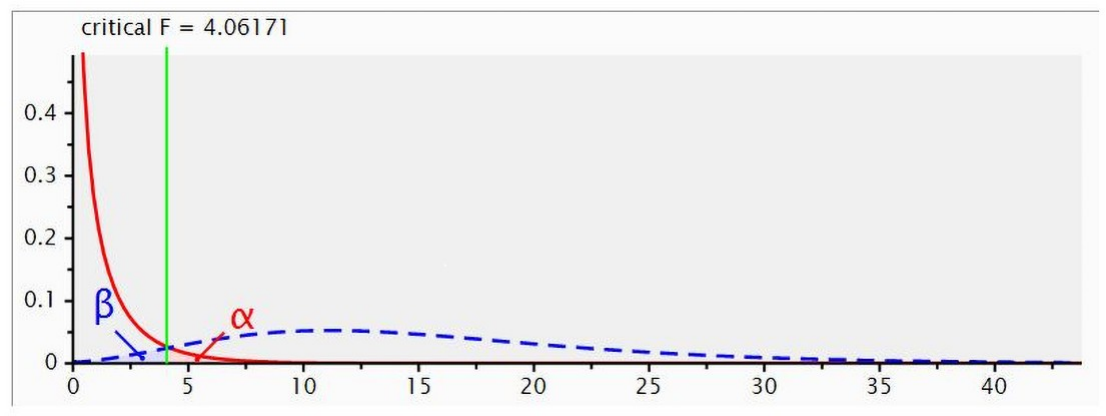
G-Power analysis for statistical power, critical F-value and sample size here.

An *a priori* power analysis was conducted using G-Power to determine the appropriate sample size (Fig 1). A total sample size of 48 steers was required to achieve statistical power of 95% with a critical F-value of 4.06 for a medium effect size and a significance level of 0.05. Therefore, forty-eight tropically adapted 24–28 months old Brahman, Charbray and Droughtmaster crossbred steers weighing 332 ± 21 kg, were used for the study. The steers were fitted with insecticidal cattle ear tags containing a synergized formulation of zeta cypermethrin (Y.Tex Corporation, Cody, Wyoming, USA) and treated with an Ivermectin based pour-on parasite control (BAYMEC, Bayer Australia Ltd, Pymble NSW, Australia) at 1 ml/10 kg liveweight (LW) dosage at the beginning of the study for internal and external parasites control. Steers were group-housed in 12 open pens (4 steers per pen) with three pens per experimental diet in a completely randomized design. Steers were allocated to four groups based on their initial liveweight to ensure similar mean liveweight per pen. The pens were then randomly assigned to one of four diets. Each pen measured 60 m^2^, fitted with 18 m^2^ shade and 4 m by 1 m feed trough. Steers were allocated to one of the following four experimental diets: 0, 15%, 30% or 45% desmanthus on DM basis with Rhodes grass hay as the basal diet. Diet CP was adjusted by the inclusion of lucerne hay in the 0, 15% and 30% desmanthus diets, based on forage CP and not DM basis, to ensure all diets were isonitrogenous. The 0 desmanthus diet (Rhodes grass and lucerne) was used as a positive control. Lucerne was selected because it is the most widely grown perennial legume globally and has been extensively studied [22]. Desmanthus consisted of the three species, namely *D*. *virgatus*, *D*. *bicornutus* and *D*. *leptophyllus* fed in equal proportions. A mixture of these three species was used because *d*esmanthus is commonly marketed as a mixed product, e.g. Jaribu desmanthus comprising *D*. *virgatus cv Marc*, *D*. *leptophyllus* cv Bayamo and *D*. *pubescens cv Uman* in the 1990s [23] and Progardes^®^ consisting of *D*. *biconutus cv JCU4*, *D*. *leptophyllus cv JCU7* and *D*. *virgatus* cv JCU2 and cv JCU5 cultivars to ensure that the best adapted cultivars eventually dominate while the less adapted cultivars take advantage of seasonal, land types and climate variation [24]. Steers were gradually adapted to the experimental diet within ten days [25]. Throughout the study, steers had unlimited access to clean water and mineral block (Table 1; Trace Element Northern, Ollson’s, Yennora, NSW, Australia).

**Table 1:** Chemical composition of the mineral block.

Ingredient	Concentration
Molasses (%)	5
Sodium chloride (NaCl, %)	82
**Macro ingredients (%)**	
Calcium (Ca)	1
Phosphorus (P)	1
Sulphur (S)	0.8
Magnesium (Mg)	0.02
**Micro ingredients (mg/kg)**	
Copper (Cu)	1000
Cobalt (Co)	65
Ferrous Iron (Fe++)	1350
Iodine (I)	500
Selenium (Se)	26
Iron (Fe)	650
Zinc (Zn)	300

Desmanthus was harvested at the late bloom to full seed maturity stages and chopped with a flail type forage harvester (New Holland model 38 Crop-Chopper, Haryana, India) every morning, while Rhodes grass and lucerne hay were chopped with a tub grinder (Roto Grind model 760, Burrows Enterprises, LLC, Greeley, CO, USA) once a week. Experimental diets were mixed by hand and offered daily between 8:30 and 9:30 am after residuals were collected. Feed offered was adjusted to allow for 5–10% refusals. Refusals were weighed daily and a sample per pen stored at -20°C, from which a weekly composite bulked sample per pen was obtained for DM and chemical analysis. Weekly samples of the desmanthus, lucerne and Rhodes grass were obtained throughout the study for DM and chemical analysis.

### Feed intake, liveweight and body condition scores

Dry matter intake (DMI) per pen was determined by the weight difference between feed offered and refusals collected 24 hrs after feeding. Steers were weighed at the start and end of the study, fortnightly for the first six weeks and monthly thereafter until the end of the study. The frequency of weighing changed after six weeks due to labour limitations resulting from COVID-19 pandemic related restrictions. All steers were weighed before feeding to reduce variation due to gut fill. Unfasted LW were recorded automatically (Gallagher 65 Scanlon Drive, Epping, Victoria 3076, Australia) and the average daily gain calculated by regressing all fortnightly and monthly LW by time in days [26]. Body condition scores (BCS) were recorded monthly using the five-point (1–5) scoring system [27].

### Forage and refusals analysis

The forage offered and refusals DM, CP, neutral detergent fibre (NDF), acid detergent fibre (ADF), hemicellulose, dry matter digestibility (DMD) nutritive values were estimated using near infrared reflectance (NIR) spectroscopy at the CSIRO Floreat laboratory (Floreat, WA, Australia). The samples were dried in a forced-air oven at 60°C for 48 h and ground to pass through a 1 mm mesh with a Christy and Norris grinder (Christy Turner Ltd, Suffolk, England), the spectra collected using the Unity Spectrastar 2500X rotating top window system (Unity Scientific, Milford, MA, USA) and predictions were generated using the chemometric software package Ucal (Unity Scientific) as described by Norman et al. [28].

### Rumen fluid collection and analysis

Rumen fluid samples were collected in the morning prior to feeding at the start, middle and end of the experimental period (week 0, 10 and 20) from 24 steers to determine the effect of diet on rumen pH, ammonia nitrogen (NH_3_-N) and volatile fatty acids (VFA). About 200 ml of rumen fluid samples were collected from the ventral sac via oro-ruminal tubing using a reinforced plastic suction tube and a hand pump. The pH of each sample was taken immediately using a portable pH meter (Aqua-pH, TPS Pty Ltd, Brendale, QLD, Australia). Rumen fluid sub-samples of 8 ml were stabilized by adding 2ml of 25% metaphosphoric acid and stored at -80°C awaiting NH_3_-N and VFA analysis. The rumen NH_3_-N was analyzed using the colorimetric method of Chaney and Marbach [29], while VFA were determined by gas chromatography (Shimadzu Corporation, Kyoto, Japan) as described by Gagen et al. [30]. Since rumen fluid with viscous appearance and pH of 7.5 or above indicate contamination with large volumes of saliva [31], samples with these characteristics were excluded in pH, total VFA and NH_3_-N analysis.

### Blood collection and plasma metabolites analysis

Blood samples were collected in the morning prior to feeding at the start, middle and end of the experimental period (weeks 0, 10 and 20) via jugular venipuncture into 10 ml sodium heparin blood Vacutainer tubes (BD, Sydney, Australia), centrifuged at 1425 x g for 20 min at 4 °C (Beckman Coulter, Inc. California, USA) to separate the plasma from the serum and stored at -80°C prior to analysis. All plasma metabolites were analyzed using the AU480 chemistry analyzer6 (Beckman Coulter, Inc. California, USA) according to the manufacturer’s procedures. Plasma non-esterified fatty acids (NEFA) were analyzed using colorimetric method [32], beta-hydroxybutyrate (BHB) and glucose analyzed by the 3-hydroxybutyrate dehydrogenase and hexokinase methods, respectively [33], total bilirubin by the modified diazo method [34] and creatinine by the kinetic modified Jaffe method [35].

### Statistical analysis

Data were analyzed using the SAS software version 9.4 (SAS Institute, Cary, North Carolina, USA), with an initial screening for data entry errors, outliers and data distribution done for all data sets. Mixed model (PROC MIXED) restricted maximum likelihood (REML) procedures in SAS fitted the fixed effect of diet and pen nested within diet as a random effect in the statistical model. Sampling week was analyzed as a repeated measure and covariance structures were specified. Final LW and BCS were analyzed by including the initial measurements as covariates. P values were deemed significant when below 0.05. When there was significant effect of diet, orthogonal polynomial contrasts were performed to test for linear, quadratic and cubic responses to increasing desmanthus proportion. The quadratic and cubic responses were dropped from the model because they were not significant for all variables tested. PROC CORR procedure fitted with Spearman’s ρ test was used to calculate the residual correlations between diet, rumen and plasma metabolite parameters. Baseline rumen and plasma metabolite values were excluded in the correlation analysis because the quality of the pasture that cattle grazed before the study commenced was not analyzed.

## Results

### Diet quality, intake and growth performance

The nutritive values of the forages are shown in Table 2 and the four experimental diets are presented in Table 3. Rhodes grass had the lowest CP and highest fibre contents compared to the legume forages. The lowest DMD and ME values were obtained in the Rhodes grass and JCU7 cultivar. The diets were formulated to be isonitrogenous hence the similar CP (*P* = 0.84). Diet NDF levels were similar (*P* = 0.40) but significant differences were observed in diets ADF, hemicellulose and ME levels (*P ≤* 0.001). There was a linear increase in ADF and decrease in hemicellulose and ME with increase in desmanthus proportion.

**Table 2:** Chemical composition (mean ± se) of the experimental diets.

Variable^1^	Rhodes grass	Lucerne	JCU2^2^	JCU4	JCU7
**Dry matter (%)**	87.9 ± 0.56	86.9 ± 0.62	31.6 ± 1.44	28.5 ± 1.25	34.3 ± 0.98
**CP**	9.0 ± 0.24	17.4 ± 0.70	13.7 ± 0.73	15.6 ± 0.61	11.9 ± 0.44
**NDF**	73.3 ± 0.40	47.6 ± 0.88	53.1 ± 1.24	50.4 ± 1.24	57.3 ± 0.75
**ADF**	42.6 ± 0.45	34.9 ± 0.86	39.6 ± 0.99	36.9 ± 0.82	41.7 ± 0.52
**Hemicellulose**	30.7 ± 0.26	12.7 ± 0.42	13.5 ± 0.84	13.5 ± 0.77	15.6 ± 0.56
**DMD**	51.8 ± 0.39	69.1 ± 1.34	55.1 ± 1.96	58.4 ± 1.58	50.6 ± 0.90
**ME (MJ/kg DM)** ^3^	7.2 ± 0.067	10.1 ± 0.23	7.7 ± 0.33	8.3 ± 0.27	6.9 ± 0.15

^1^CP: crude protein, NDF: neutral detergent fibre, ADF: acid detergent fibre; DMD: dry matter digestibility. Data are presented in percentage of dry matter (DM) unless otherwise stated.

^**2**^JCU2: *D*. *virgatus*, JCU4: *D*. *bicornutus*, JCU7: *D*. *leptophyllus*.

^**3**^Estimated from *in vitro* dry matter digestibility as DMD × 0.172–1.707 [36].

**Table 3:** Effect of desmanthus proportion on the nutritive value, digestibility and dry matter digestibility to crude protein ratio over the feeding trial duration.

	% desmanthus in the diet					Linear *P*-value
Variable^1^	0	15	30	45	SEM^2^	
**CP**	11.6	11.6	11.5	11.4	0.11	0.84
**NDF**	65.2	64.9	64.6	64.4	0.15	0.40
**ADF**	40.1	40.4	40.8	41.1	0.078	0.0007
**Hemicellulose**	25.1	24.4	23.8	23.3	0.14	0.001
**ME (MJ/kg)** ^3^	8.1	7.9	7.6	7.4	0.032	<.0001
**DMD:CP ratio**	8.7	8.5	8.5	8.5	0.093	0.91

^1^CP: crude protein, NDF: neutral detergent fibre, ADF: acid detergent fibre, DMD: dry matter digestibility; Values are presented in least square means. Data are presented in percentage of dry matter (DM) unless otherwise stated.

^2^SEM: standard error of the mean.

^3^Estimated from individual forages *in vitro* DMD as DM digestibility × 0.172–1.707 [36].

The DMI and nutrient intake data are presented in Table 4. Increasing the desmanthus levels in the diet decreased the DMI and subsequently CP, NDF, ADF, hemicellulose and ME intake (*P* ≤ 0.008). The DMI decreased from 8.8 to 7.6 kg/day with increasing desmanthus proportion from 0 to 45% (*P* = 0.009).

**Table 4:** Effect of desmanthus proportion on nutrient intake.

	% desmanthus in the diet					Linear *P*-value
Variable^1^	0	15	30	45	SEM^2^	
**DM**	8.8	8.5	8.2	7.6	0.061	0.009
**CP**	1.0	1.0	0.95	0.89	0.011	0.007
**NDF**	5.7	5.4	5.2	4.8	0.044	0.003
**ADF**	3.5	3.3	3.2	3.0	0.025	0.008
**Hemicellulose**	2.1	2.0	1.9	1.8	0.021	0.001
**MEI (MJ/day)**	72.8	69.8	66.2	61.7	0.49	0.001

^1^Data are presented in kg/day unless otherwise stated. DM: dry matter, CP: crude protein, NDF: neutral detergent fibre, ADF: acid detergent fibre, MEI: metabolizable energy intake. Values are presented as least square means.

^2^SEM: standard error of the mean.

There were no significant differences in LW, BCS, ADG and feed to gain ratio observed between diets (Table 5). At the end of the study steers weighed between 419 to 434 kg (*P* = 0.21) with feed to gain ratio of 12.9 to 14.6 kg DMI per kg weight gained (*P* = 0.31).

**Table 5:** Effect of desmanthus proportion on the average liveweight, body condition score, daily gain and feed to gain ratio in supplemented steers.

	% desmanthus in the diet					
Variable^1^	0	15	30	45	SEM^2^	*P*-value
**Overall liveweight (kg)**	392.6	388.0	384.5	381.3	1.98	0.28
**Final liveweight (kg)**	434.0	439.5	426.7	419.3	4.49	0.21
**Final Body condition score**	3.3	3.4	3.3	3.2	0.051	0.33
**Average daily gain (kg/day)**	0.62	0.66	0.57	0.52	0.040	0.13
**Feed:Gain ratio**	14.2	12.9	14.6	14.5	0.35	0.31

^1^ Values are presented as least square means.

^2^SEM: standard error of the mean.

### Rumen and plasma metabolite parameters

Rumen pH and metabolites data of steers fed increasing desmanthus level*s* in the diet are presented in Table 6. A linear decrease in total VFA with incremental levels of desmanthus in the diet was observed (*P* = 0.026). The rumen pH and all the other metabolites measured were similar for all the diets (*P* ≥ 0.076). Proportion of desmanthus in the diet had no effect on plasma NEFA, BHB, glucose, creatinine and total bilirubin (Table 7; *P* ≥ 0.067).

**Table 6:** Rumen volatile fatty acids (VFA), ammonia nitrogen (NH3-N) and pH of tropical beef cattle fed increasing levels of desmanthus.

	% desmanthus in the diet					
Variable^1^	0	15	30	45	SEM^2^	*P*-value
**Total VFA (mg/100dL)**	68.3	59.2	61.7	57.5	1.53	0.026
**Acetate (molar %)**	74.1	74.0	73.3	73.8	0.18	0.49
**Propionate (molar %)**	14.8	14.1	14.7	14.2	0.15	0.32
**Acetate/Propionate ratio**	5.0	5.2	5.0	5.2	0.061	0.37
**Iso-butyrate (molar %)**	1.4	1.5	1.3	1.4	0.056	0.63
**n-butyrate (molar %)**	6.6	7.1	7.2	7.3	0.093	0.083
**Iso-valerate (molar %)**	1.3	1.4	1.2	1.4	0.040	0.41
**n-valerate (molar %)**	0.60	0.65	0.66	0.66	0.009	0.076
**n-caproate (molar %)**	0.85	1.0	1.0	1.0	0.038	0.12
**NH**_**3**_ **-N (mg/dL)**	12.2	11.6	11.6	10.4	0.349	0.31
**pH**	6.9	7.1	7.1	7.1	6.58^3^	0.20

^1^ Values are least square means. VFA: volatile fatty acids, NH_3_-N: ammonia nitrogen.

^2^SEM: standard error of the mean.

^3^SEM presented as H^+^ concentration in nEq/L.

**Table 7:** Plasma metabolites of tropical beef cattle fed increasing levels of desmanthus.

	% desmanthus in the diet					
Metabolite^1^	0	15	30	45	SEM^2^	*P*-value
**NEFA (mmol/L)**	0.52	0.40	0.44	0.50	0.029	0.92
**BHB (mmol/L)**	0.23	0.24	0.27	0.21	0.006	0.067
**Glucose (mmol/L)**	5.1	5.2	5.1	5.1	0.075	0.99
**Creatinine (μmol/L)**	108.3	106.8	109.1	113.7	1.41	0.41
**Total bilirubin (μmol/L)**	3.2	3.0	3.0	3.3	0.10	0.67

^1^ Values are least square means. NEFA: non-esterified fatty acids, BHB: β-hydroxybutyrate.

^2^SEM: standard error of the mean.

Medium to high residual correlations (0.41–0.83) were observed between diet parameters, rumen VFA and plasma metabolites (Table 8). Diet ADF was correlated to all rumen metabolites except NH_3_-N and n-caproate. A negative correlation between diet ADF with total bilirubin and NEFA was observed. Diet CP correlated positively with rumen NH_3_-N and propionate but negatively with acetate/propionate ratio, n-caproate and creatinine (*P* < 0.05).

**Table 8:** Residual correlation coefficients between diet, rumen and plasma metabolite parameters.

	Diet parameters^2^				
Metabolites^1^	CP	NDF	ADF	Hem	DMD
**Total VFA**	0.20	0.65**	-0.68**	0.65**	0.51**
**A/P ratio**	-0.58**	0.41*	-0.56**	0.55**	-0.053
**NH**_**3**_ **-N**	0.63**	0.17	-0.026	0.068	0.46*
**Acetate**	-0.30	0.62**	-0.79***	0.72***	0.25
**Propionate**	0.64**	-0.3	0.45*	-0.45*	0.16
**Iso-butyrate**	0.26	-0.60**	0.64**	-0.66**	-0.21
**n-butyrate**	-0.17	-0.4	0.41*	-0.36	-0.38
**Iso-valerate**	0.034	-0.69**	0.72***	0.73***	-0.44*
**n-valerate**	-0.0026	-0.73***	0.83***	-0.79***	-0.49*
**n-caproate**	-0.51*	-0.11	0.072	-0.046	-0.37
**Total bilirubin**	-0.25	0.58**	-0.62**	0.66**	0.17
**Creatinine**	-0.59**	0.092	-0.26	0.22	-0.24
**Glucose**	0.11	0.42*	-0.36	0.43*	0.30
**BHB**	-0.07	0.15	-0.16	0.19	0.066
**NEFA**	-0.37	0.39	-0.45*	0.51*	0.00

^1^VFA: volatile fatty acids, A/P ratio: acetate to propionate ratio, NH_3_-N: ammonia nitrogen, NEFA: non-esterified fatty acids, BHB: β-hydroxybutyrate.

^2^DM: dry matter; CP: crude protein; NDF: neutral detergent fibre; ADF: acid detergent fibre, Hem: Hemicellulose, DMD: dry matter digestibility.

* = *P* < 0.05,

** = *P* < 0.01,

*** = *P* < 0.001.

## Discussion

This study evaluated the effect of incremental levels of desmanthus in isonitrogenous diets on the feed intake, rumen fermentation, plasma metabolites and growth rate of tropical crossbred beef steers.

### Diet quality, intake and growth performance

Rhodes grass and lucerne quality indicators were within the range reported in other studies [37–40]. Rhodes grass had lower CP and higher fibre content compared to legume forages. The observed difference agrees with previous studies that had reported grass to have lower quality than legume forages [38, 41]. The CP value of *D*. *virgatus* and *D*. *bicornutus* were within the values reported in previous studies carried out in northern Australia [17, 42, 43]. The CP content for *D*. *leptophyllus* was comparatively lower than the CP of *D*. *virgatus* and *D*. *bicornutus* [17, 42] but similar to that reported by Suybeng et al. [17]. Differences in values between studies may be due to soil fertility, climate, plant fraction and stage of maturity at harvest [44]. The quality differences between species may be due to differences in plant characteristics. For instance, desmanthus ranges widely from early to late maturing and herbaceous to suffruticose plant types [24, 43]. The fibre content was lower than that reported by Suybeng et al. [17] but higher than that reported by Durmic et al. [42]. This difference may be due to the maturity stage and plant part collected. Unlike this study where the whole plant was used, Durmic et al. [42] collected the leaves and only 5cm long stems to mimic cattle grazing. Significant variation in plant part nutritive value has been widely reported [45, 46].

Diets were formulated to ensure that they were isonitrogenous but increasing desmanthus proportion in the diet increased ADF content and reduced NDF and DM intake. High indigestible fibre content reduces voluntary feed intake in ruminants due to low digestibility and high rumen fill effect [47, 48]. Dairy cows fed grass silage of varying maturity were observed to eat less as the ADF content increased [49]. Dietary fibre content has been widely used to predict digestibility. A negative correlation exists between indigestible fibre content and digestibility [50, 51]. However, DMD is reported to restrict rumen fermentation and DM intake when the DMD:CP ratio exceeds 8 to 10 [52], but in this study, the ratio ranged between 8.5 and 8.7 indicating that DMD was not likely to limit rumen fermentation and feed intake.

Confined *Bos indicus* crossbred beef cattle weighing 400kg fed an 8 MJ/kg DM diet require 40 MJ ME daily for maintenance and 69 MJ ME for 0.5 kg ADG [53]. In this study, the crossbred steers consumed 61–72 MJ ME daily; hence weight gain was expected. Steers in all experimental diets gained at least 0.5 kg/day. Typically, the annual ADG of cattle grazing northern Australian native grass pastures is 0.3 kg/day [3, 8]. Poppi et al. [3] reported that ADG ranging between 0.4 and 0.6 kg can be achieved using improved forage, and these gains are adequate to meet the target slaughter weight required for the prime beef market within 2.5 years of age. The observed significant drop in feed intake as the desmanthus level increased did not result in a significant difference in LW. The reduced feed intake may have improved digestibility by reducing the passage rate of digesta from the reticulorumen [54]. Besides, desmanthus is a tannin-containing legume [17, 43]. Condensed tannins can form complexes with proteins that protect CP from microbial degradation in the rumen, thus enhancing lower gastrointestinal tract digestion [55]. Lack of significant difference between diets indicates that desmanthus alone or mixed with other high-quality legume forages can be used to supplement grass-based diets of beef cattle in northern Australia without any detrimental impact on productivity. Supplementation of grass-based grazing cattle with desmanthus was reported to improve growth rate [15]. In contrast to our findings, incremental levels of up to 31% desmanthus resulted in only 0.2 kg ADG in confined steers even when 11.8% dietary CP was achieved [17]. The authors attributed the low weight gain to low DM intake. The steers consumed 1.4% DM/LW compared to at least 2% in this study. The feed to gain ratio in this study (12–14) was similar to values reported for cattle fed high forage diets of similar CP. Continental crossbred steers fed a 70:30 forage to concentrate diet with 13% CP had feed to gain ratio of 10.4–16.9 [56], while *Bos indicus-Bos taurus* crossbred cattle fed 11% CP diet consisting of 5–24% concentrate supplement had 9.4–13.5 feed to gain ratio [55].

### Rumen and plasma metabolites

VFAs play a major role in ruminant nutrition [57]. They contribute 60–70% of metabolizable energy [58, 59]. The VFA levels vary with animal feeding patterns and diet composition. Highly fermentable diets may raise rumen VFA levels to 200 mM, with the peak occurring 2 to 4 h after feeding. However, hay diets produce smaller fluctuations throughout the day and concentrations of less than 100 mM are common [60]. Previous studies reported increases in total rumen VFA with increases in diet digestibility [49, 61]. This agrees with our findings of a high negative correlation between ADF, a marker of diet digestibility, and total VFA. Total VFA concentrations decreased with an increase in desmanthus level in the diet. This could be due to several factors: a) The decrease in DMI associated with higher proportion of desmanthus in the diet increased ADF and reduced feed intake [58]. The increase in ADF was associated with a decrease in total VFA concentration, in line with previous observations in a dual-flow continuous culture system where high levels of indigestible fibre resulted in low concentrations of rapidly fermentable carbohydrates and reduced VFA concentration [62]. b) Condensed tannins may have formed complexes with proteins availing proteins for digestion in the lower gastrointestinal tract [55]. This may increase microbial protein synthesis efficiency in the rumen [63]. On the other hand, lucerne is highly fermentable in the rumen [64], hence the high total VFA. Rumen NH_3_-N levels of 10 mg/dl are required to maintain effective rumen microbial activity and maximize voluntary DMI in cattle fed low-quality tropical forage [65, 66]. These levels (10.4–12.2 mg/dl) were attained in all the diets in this study.

The molar proportion of individual VFA is influenced by dietary composition and DMI [62]. An increase in the proportion of dietary forage or fibre leads to an increase in acetate and decrease in propionate, butyrate, iso-butyrate, valerate and iso-valerate molar concentrations [49, 67]. Similar to our study, cattle grazing pasture of varying digestibility had similar rumen molar percentages of acetate, propionate, butyrate, iso-valerate and valerate [68]. However, in contrast to our study, they reported an increase in iso-butyrate concentrations with increased diet DMD. However, the increase was numerically small and considered to be of no biological significance.

All plasma metabolites measured in this study were not affected by the dietary desmanthus levels. Cattle fed diets of similar CP as in this study had similar glucose and NEFA concentrations [69]. Plasma NEFA originates from the mobilisation of stored fat. The similar NEFA concentration between diets may indicate that the steers had comparable energy balance [70]. In ruminants, negligible levels of glucose are absorbed across the portal-drained viscera from dietary sources. Propionate plays a major role as a precursor for glucose synthesis, contributing 46–73% to hepatic gluconeogenesis in cattle [57]. The similar molar proportion of propionate in the current study may have resulted to similar glucose levels between the diets. All plasma metabolites measured were within the normal range reported for beef cattle [71–74]. Similar levels of plasma metabolites indicate that an inclusion level of up to 45% desmanthus in the diet does not cause adverse effects on energy metabolism and health status of supplemented steers.

Overall, increasing the proportion of desmanthus in the diet did not influence LW, ADG, VFA molar proportion and plasma metabolites of steers fed isonitrogenous diets. These findings indicate that desmanthus can be used as a renewable protein source for beef cattle in the northern Australian beef cattle production system, particularly in the tropical semi-arid clay soil environments where lucerne is not adapted.

The northern Australian beef industry serves both the pasture-fed and grain-finished beef markets [1]. It is common practice for cattle backgrounded on pastures to be finished in the feedlot on energy-dense grain diets for short periods of about 100 days before slaughter [75]. Further studies are required to determine the effect of desmanthus supplementation on feedlot performance and carcass quality of both pasture and feedlot finished cattle.

## Conclusion

This study aimed to evaluate the weight gain, rumen fermentation and plasma metabolites of tropical crossbred steers in response to supplementation with incremental levels of desmanthus forage legume in isonitrogenous diets. The results showed similar weight gains, VFA molar proportion and plasma metabolites, but a decrease in total VFA with increase in dietary desmanthus levels. Hence, the hypothesis that cattle fed isonitrogenous diets supplemented with different desmanthus level*s* will have similar growth rates, rumen fermentation and plasma metabolites was accepted. The results indicate that desmanthus alone or in combination with other high-quality legume forages can be used to supplement grass-based diets of beef cattle in northern Australia. However, more studies are required to examine the effect of desmanthus supplementation on cattle feedlot performance and carcass quality.
